# Gender-specific predictors of at-risk adolescents’ hazardous alcohol use—a cohort study

**DOI:** 10.1186/s13011-017-0105-6

**Published:** 2017-05-15

**Authors:** Camilla Jalling, Tobias H. Elgán, Anders Tengström, Andreas Birgegård

**Affiliations:** 1 STAD, Centre for Psychiatry Research, Department of Clinical Neuroscience, Karolinska Institutet, & Stockholm Health Care Services, Stockholm County Council, Norra Stationsgatan 69, SE-113 64 Stockholm, Sweden; 2 KÄTS, Centre for Psychiatry Research, Department of Clinical Neuroscience, Karolinska Institutet, & Stockholm Health Care Services, Stockholm County Council, Norra Stationsgatan 69, SE-113 64 Stockholm, Sweden; 3 Centre for Psychiatry Research, Department of Clinical Neuroscience, Karolinska Institutet, & Stockholm Health Care Services, Stockholm County Council, Norra Stationsgatan 69, SE-113 64 Stockholm, Sweden

**Keywords:** Heavy episodic drinking, Risk-use of alcohol, Externalizing behavior, Adolescents, Peers, Problem-behavior theory

## Abstract

**Background:**

Previous research has found strong associations between adolescents’ hazardous alcohol use and their perception of peer behavior, as well as own spending money and a range of antisocial behaviors. However, there is insufficient evidence of gender-specific predictors among adolescents with elevated antisocial behavior and alcohol use to design effective selective interventions. The aims of this study were to test short-term predictors of Heavy Episodic Drinking (HED) and risk-use of alcohol among 12-18-year-old females and males with elevated externalizing and delinquent behavior, and alcohol use.

**Methods:**

Eighty-five females, 77 males, and their parents, originally recruited for a parent intervention, were assessed at baseline and 6 months later with several validated instruments measuring externalizing and internalizing behavior, alcohol use, psychosocial distress, and delinquency.

**Results:**

The perception of peer drinking significantly predicted both genders’ HED and risk-use, and also externalizing behavior predicted female risk-use. Rule-breaking behavior and social problems predicted both HED and risk-use among males, while rule-breaking predicted female HED and social problems predicted female risk-use. The parents’ ratings of externalizing behavior predicted only their sons’ risk-use. Lastly, no differences in prediction strength were found to be statistically significant differences between genders.

**Conclusions:**

Females and males shared several predictors of hazardous alcohol use, and perception of peer drinking emerged as a strong predictor. This suggests that interventions may target both genders’ hazardous use of alcohol, and should address peer-resisting skills.

## Background

Alcohol initiation typically occurs during adolescence, and drinking patterns and the consequences thereof in the adolescent age group have been extensively studied. For example, early drinking onset, drinking several days a week, and drinking larger quantities at single occasions are factors that define at-risk populations in that they increase the risk of developing a range of short-term problems that may persist or increase in adulthood [1]. In fact, alcohol use during adolescence has been found to be among the largest risk factors for disease development [2, 3], and also to have medical and psychological consequences [4, 5]. Furthermore, heavy alcohol use has for some time been recognized to be associated with a range of adverse short-term outcomes [3, 6–10]. The increased risk of such adverse outcomes makes it imperative to explore the mechanisms behind adolescents’ hazardous use of alcohol in order to develop effective interventions.

Underscoring the need to include variables from several domains simultaneously to understand problem behavior in adolescents, Richard Jessor [11] describes Problem-Behavior Theory (PBT), where adolescent drinking and other problem behaviors are interrelated because of a general proneness to deviant behavior. Jessor [11] proposes different systems that psychosocially influence each other – personality, perceived environment, and behavior systems. The joint influence that these systems have on behavior problems determines the level of proneness to normative transgressions or problem behavior. A body of research has confirmed such relationships (for instance [12, 13]), and associations have been found between hazardous drinking and a range of both internalizing problems [10, 14–16], externalizing problems [16–18] including delinquency [19–21], and psychosocial problems [12]. Gender differences have, however, been suggested [13].

Jessor [1] further proposes in PBT theory that the social environment influences problem behavior, and specifically that adolescent drinking is affected by an adolescent’s deviant peers. Research has shown alcohol-using peers to be one of the most consistent predictors of adolescents’ drinking [22]. This has in some studies been found to affect females more than males [22, 23], and older adolescents more than younger [24]. The environmental system also includes parental support and control [11]. Perceived parental knowledge, i.e., if adolescents think their parents have insight into their activities, has been found to relate to less adolescent alcohol use and delinquency [25]. Less is known, however, about parental perception of adolescents’ psychosocial status and antisocial behaviors, and its associations with adolescent female and male alcohol use.

The present study contributes to the research field with results from a naturalistic community sample of adolescents: undiagnosed, but with enhanced levels of problem behaviors, which puts them at risk for the aforementioned adversities. In an attempt to prevent further problem-behavior development with regards to hazardous alcohol use and externalizing behavior, the parents of the adolescents received a parent-training intervention. However, an evaluation revealed a null effect [explained in 26], which warranted further exploration of the adolescents’ possible predictors of hazardous use of alcohol.

In order to understand how adverse behaviors affect consumption quantity and related problems, delimitations have been defined. Heavy episodic drinking, HED, is here defined as having six or more drinks at a single occasion. The definition is similar to binge drinking, which is usually defined with a time window of 2 h, and four drinks for women or five for men [26]. In the following text we also use the term risk-use of alcohol, which concerns the amount of alcohol consumed (i.e., quantity and frequency), the risk for alcohol-related physical and psychological harm, and dependency. The reason to use both HED and risk-use is that the former refers to the quantity at a single drinking occasion, which indicates immediate short-term risk of alcohol-related adversities, while the latter indicates long-term alcohol-related consequences.

Previous research has employed cross-sectional or longitudinal designs with long-term follow-ups. However, the research field suffers a lack of additional knowledge with regards to short-term prediction studies aimed at understanding whether adolescents’ antisocial behaviors and psychosocial problems may predict drinking behavior. The results from the present study may illuminate how different aspects of at-risk adolescents’ behaviors can be addressed in interventions, in order to decrease future hazardous alcohol use.

The aims of the current study were 1. to test antisocial behaviors and psychosocial problems as predictors for HED and risk-use, 2. to test if parental ratings of the adolescents’ antisocial behaviors predicted the adolescents’ HED and risk-use, and 3. to explore if the adolescents’ monthly spending money, perceived family economic standard, delinquent and drinking peers, parental country of birth, gender, and/or age, predicted HED and risk-use of alcohol. As previously noted, research suggests that females can be more sensitive to peer drinking, and we therefore hypothesized that perceived peer drinking would have a larger effect on females’ HED and risk-use than on males’.

## Methods

### Participants and recruitment

This cohort study uses a six-month follow-up design and consists of 85 female and 77 male alcohol-using adolescents: a naturalistic subsample of 67% out of 243 adolescents who, along with their parents, were originally recruited for a study on two parent interventions taking place in Stockholm County between the years 2008 and 2010. Eligible participants to the intervention trial were parents and their 12-18-year-olds who were at risk of consolidating their already elevated antisocial behavior. Results from the study revealed that none of the two interventions had effect on adolescent antisocial behavior, delinquency, alcohol use or psychosocial distress when compared with the control group (for further description of interventions and results see [27]).

### Assessments

The adolescents and parents were assessed at baseline and at a six-month follow-up through paper questionnaires for parents and web-based questionnaires for adolescents. Due to the original randomized controlled trial (RCT) design, inclusion in the study and data collection was conducted in four waves, with the first baseline assessment in the fall of 2008 and the last six-month follow-up in late spring 2010.

### Instruments

#### Alcohol use disorder identification test (AUDIT)

The AUDIT was used to measure self-reported alcohol risk-use and HED. The scale consists of 10 items, with the total score ranging from 0 to 40 points [28]. The AUDIT has in several studies been confirmed to be psychometrically valid in multiple adult populations, as shown in the reviews of de Meneses-Gaya, Zuardi [29] and Pilowsky and Wu [30]. Among adults, a total score of ≥ 8 indicates risk-use, i.e., hazardous drinking, harmful drinking and possible dependency [31]. A few validation studies on adolescent samples have also been conducted, conveying a psychometrically sound AUDIT total scale and subscales in different populations [32–35]. Different thresholds of adolescent risk-use have been discussed, varying between 2 and 11 points; in accordance with a recent German study we used a cut-off point of ≥ 6 points for both female and male adolescents’ risk-use [33]. Additionally, item 3 (“How often do you have six or more drinks at one occasion?”) was used to measure HED with a cut-off score of ≥ 2 points, i.e., “Monthly” or more often [31]. It should be noted that some studies have defined HED as “*five* or more drinks at one occasion during the last 30 days” [23, 25, 36–38], while the AUDIT item asks about *six* drinks or more. This implies that our risk-using subsample will be at slightly greater risk than samples in some other studies. Lastly, it should be noted that when assessing HED, no differential gender threshold was applied.

#### Child behavior checklist & youth self-report (CBCL & YSR)

Achenbach’s well-validated scale CBCL and YSR, which includes externalizing and internalizing broadband scales, were used. Items are rated on a 3-point scale: 0 (never/seldom); 1 (sometimes); 2 (often/always). The internalizing broadband scale taps anxiety, withdrawnness, and somatic complaints and ranges from 0 to 62 points, while the externalizing broadband scale (rule-breaking and aggressive behavior) ranges from 0 to 64 points.

#### Self-reported delinquency (SRD)

The total scale score (excluding the subscale Hard drug use) was used to measure overt and covert behaviors that tap violence, general delinquency, and status offenses. Adolescents were asked how many times they had performed any of the 40 behaviors on the list during the last 6 months, rated from 0 to “9 times or more.” The total score is 0-360 points [39].

#### Youth-outcome questionnaire self-report (Y-OQ® 2.0 & Y-OQ®SR 2.0)

Parent and adolescent questionnaires rate adolescents’ psychosocial distress and treatment progress. Sixty-four items are rated on a 5-point scale (range 0-4), including eight reversed items that tap healthy behaviors. The Y-OQ® covers six domains: Intrapersonal distress (anxiety, depression, hopelessness); Somatic complaints (headaches, dizziness, stomach aches); Interpersonal relations (arguing, defiance, communication problems); Social problems (delinquent or aggressive behaviors); Behavioral dysfunction (organization, concentration, handling frustration, and ADHD-related symptoms); and Critical items, i.e., ”…symptoms often found in youth receiving inpatient services, such as paranoid ideation, hallucinations, mania, and suicidal feelings” ([40], page 1117).

#### Delinquent & drinking friends

There were five questions about delinquent friends: “How many of your friends: use alcohol regularly; use illicit drugs; commit property crimes; fight physically; hang around in the city at night?” The answers are rated on a 4-point scale (1 = No one; 2 = Maybe someone; 3 = A few; 4 = Most of them), and the total sum ranged from 5 to 20 points. Item 1 (“How many of your friends: use alcohol regularly?”) was also used as a separate categorical predictor variable, albeit dichotomized, using the merged responses 1 − 2, and 3 − 4.

### Missing data

The present study extracted a sub-sample of drinking adolescents and their parents, only. In the original study to which they were recruited, the proportion of missing data in parental responses was considered to be small (from 0.4 to 3.5% on single items). It was handled with the single imputation method EM (estimation-maximization algorithm), using Little’s MCAR test, which revealed that the missing values were missing at random (MAR) [41]. Dyads were omitted from this study if any of them were lost to follow-up, which in this sample of self-reported drinking adolescents only occurred with 1.2% (*n* = 1).

### Statistical analyses

In the analyses, all predictors were based on the baseline values. The outcomes, HED and risk-use, were based on the follow-up measurement using the aforementioned cut-off values (see Methods/Instruments). Further, in order to minimize Type 1 error, all analyses (with exception for multiple regression correlation (MRC) analyses, see below) have been adjusted for multiple testing using an α at ≤ .010 for statistical significance. This entails that the confidence intervals, CI, were adjusted to 99%. Firstly, descriptive statistics were examined, and mean values (*M*) and standard deviations (*SD*) for all predictors and outcomes at baseline were assessed. Tests for gender mean differences in the predictors at baseline (T1) and follow-up (T2) were conducted using *t-tests.*

Predictor variables were then tested for linearity with the logit of the outcome. This was done to avoid over-dispersion, i.e., that the variance in the logistic model is larger than expected and OR too big, which may lead to Type 1 errors [42]. The analyses were conducted with two logistic regression models – one each for the outcomes HED and risk-use. In each model, all predictors were entered, and also their natural logarithm*predictor interaction term, and the binary outcome as a dependent variable. Predictors with significant interaction terms failed to meet the linearity assumption and were not entered into subsequent prediction models.

Exploratory logistic regressions, using the stepwise method with backward likelihood ratio elimination as suggested by Field [42], were performed to study if females’ and males’ problem behavior and delinquent friends predicted HED and risk-use of alcohol. This method was chosen due to the study’s exploratory nature, in order to find the most important predictors of HED and risk-use. Predictor variables in the logistic regression models were seven at most, thus not violating “the rule of thumb” suggesting a minimum of ten events per predictor. Also, research has shown that observations of less than ten events per predictor variable should not entail a risk of bias or Type 1 errors [43]. In fact, Vittinghoff and McCulloch [43] suggest that when a significant association in a logistic regression is found and when the number of events per variable is close to five, only a minor degree of caution is justified. Since the present sample is a subsample of adolescent alcohol users, we did not control for alcohol use baseline scores in the regression models, due to the risk of bias and Type 2 errors [44, 45].

For predictors that were significant in either gender, we performed direct tests of gender differences in prediction slopes using multiple regression correlation (MRC) analyses, using an α level of .05. Each model comprised the binary gender variable, a *z*-transformed predictor score, and the *z*-transformed predictor*gender interaction term.

To further explore more specific and clinically meaningful predictors, the subscales that compose the externalizing and internalizing scales, as well as psychosocial distress (as measured with Y-OQ®SR), were tested using logistic stepwise regression, with the backward likelihood ratio method. CIs were set to 95%.

Chi-square analysis was performed to assess the relationship between parental country of birth, adolescents’ monthly spending money, perceived family economic standard, and risk-use and HED. Initially, we tested if the intervention group alone, or in interaction with gender, affected alcohol use at baseline, which it did not. In subsequent analyses, intervention was left out and gender was used to split the sample.

## Results

### Background characteristics

Table 1 shows proportions of adolescent females’ and males’ characteristics. Female HED and risk-use proportions were slightly higher than male HED and risk-use. Markedly fewer of the females’ mothers were born outside the Nordic countries, and the females also reported a markedly larger proportion of drinking friends.

**Table 1:** Background characteristics, given in percentages

		Females	Males
Gender proportion		52.5	47.5
Born outside the Nordic countries	Mother	8.2	26.0
	Father	29.4	28.6
Living all their life in Sweden		90.6	89.6
Heavy episodic drinkers		45.9	36.4
- weekly or almost daily		20.1	14.3
Risk drinkers		69.4	61.0
Some or most friends drink alcohol		67.1	45.4

Females *n* = 85, males *n* = 77

Table 2 shows the mean values of females and males, and the results from *t*-tests for gender differences on the predictors and outcome variables at T1 and T2. As shown, the mean externalizing behavior at T1, internalizing behavior at T1 and T2, and psychosocial problems at T1 and T2 were significantly different between genders. The females rating of their perception of delinquent friends at T1 was close to significantly higher than males, i.e., *p* = .011, and drinking friends at T2 showed *p* = .013. On the outcomes HED and risk-use, there were no significant gender differences. Parental ratings of their child showed significant gender differences on T1 internalizing behavior only.

**Table 2:** Baseline and 6-month follow-up mean values of the predictors and outcomes, along with the standard deviation (*SD*). Results from t-tests examining gender differences, and Cronbach’s α for scale reliability

	Time point	Female mean (*SD*)	Male mean (*SD*)	*t-*value *(df = 160)*	*p value*	Cronbach’s α
Adolescent self-rated						
Outcomes						
Heavy episodic drinking, HED	T1	0.72 (0.96)	0.60 (0.75)	0.88	.378	NA
	T2	1.48 (1.04)	1.23 (1.05)	1.51	.133	NA
Risk-use of alcohol	T1	5.22 (6.17)	4.21 (5.99)	1.06	.761	0.76
	T2	8.54 (7.33)	7.61 (8.03)	0.77	.659	0.83
Predictor*s*						
Age	T1	14.86 (1.67)	15.31 (1.62)	−1.75	.083	NA
Externalizing behavior	T1	22.01 (9.18)	17.33 (8.47)	3.63	< .001	0.87
	T2	21.59 (9.92)	17.79 (10.97)	2.31	.022	0.91
Internalizing behavior	T1	9.78 (6.27)	6.05 (5.27)	4.07	< .001	0.88
	T2	14.59 (9.35)	9.87 (9.43)	3.20	.002	0.91
SRD	T1	42.99 (39.89)	38.23 (42.12)	0.74	.462	0.93
	T2	44.85 (43.89)	40.91 (55.03)	0.51	.613	0.95
Y-OQ®	T1	60.39 (30.22)	43.79 (28.37)	3.59	< .001	0.93
	T2	54.62 (30.97)	41.40 (32.50)	2.65	.009	0.94
Drinking friends	T1	3.05 (1.01)	2.60 (1.27)	1.79	.074	NA
	T2	3.05 (1.01)	2.73 (1.25)	2.50	.013	NA
Delinquent friends	T1	11.43 (4.14)	9.66 (4.64)	2.57	.011	0.82
	T2	12.03 (4.55)	10.78 (4.93)	1.68	.094	0.87
Parent-rated						
Externalizing behavior	T1	21.89 (9.93)	20.08 (8.83)	1.21	.226	0.86
	T2	15.08 (11.50)	11.74 (8.97)	2.05	.042	0.90
Internalizing behavior	T1	14.71 (9.54)	10.39 (7.59)	3.17	.002	0.88
	T2	8.52 (8.43)	6.29 (6.79)	1.84	.068	0.90
Y-OQ®	T1	60.09 (32.13)	55.23 (26.97)	1.04	.301	0.92
	T2	50.94 (32.49)	41.69 (27.40)	1.95	.053	0.94

T1 = baseline measurement, T2 = 6-month follow-up measurement. *SRD* - self-reported delinquency, *Y-OQ®* - youth outcome questionnaire®. Externalizing and Internalizing behavior was measured using Child Behavior Checklist questionnaire (CBCL). Results of risk-use is the mean of all values above the cut-off for risk-use, i.e., >6 points. Similarly, HED consists of mean above the cut-off >2 points. Due to multiple testing the α level for significance was set to .01

### Tests of predictors

The results from tests for linearity of the logit regressions showed significant interaction terms for internalizing and externalizing behavior when males’ risk-use was the outcome. These scales were thus omitted from the subsequent prediction analysis model.

### Tests of prediction of HED and risk-use

As shown in Table 3, drinking friends emerged as a consistent predictor of adolescent problem drinking behavior. Female risk-use was also predicted by their externalizing behavior. The Nagelkerke pseudo *R* ^2^ showed acceptable account for the models’ total variance (between 22 and 38%). Parents’ ratings of externalizing behavior predicted their sons’ risk-use, but adjusted *R* ^2^ that the model accounted for limited variance (Table 3).

**Table 3:** Binary logistic regressions backward log likelihood method analyses for prediction of females’ and males’ HED and risk-use

	Female HED				Male HED				Female risk-use				Male risk-use			
*self-rated*	*OR*	*p*	*CI*	*R*_*N*_^*2*^	*OR*	*p*	*CI*	*R*_*N*_^*2*^	*OR*	*p*	*CI*	*R*_*N*_^*2*^	*OR*	*p*	*CI*	*R*_*N*_^*2*^
				0.27				0.36				0.38				0.22
Age	-	-	-		-	-	-		-	-	-		-	-	-	
Externalizing behavior	-	-	-		-	-	-		1.18	.003	1.02, 1.36		NA	NA	NA	
Internalizing behavior	-	-	-		-	-	-		-	-	-		NA	NA	NA	
SRD	-	-	-		-	-	-		-	-	-		-	-	-	
Y-OQ*®*	-	-	-		-	-	-		-	-	-		-	-	-	
Drinking friends (cat)	7.59	.003	1.30, 44.44		9.88	≤.001	2.01, 48.59		5.72	.004	1.21, 26.97		4.56	.003	1.20, 17.36	
Delinquent friends	-	-	-		-	-	-		-	-	-		-	-	-	
*parent-rated*																
				NA								NA				0.14
Externalizing behavior	-	-	-		-	-	-		-	-	-		1.09	.007	1.00, 1.19	
Internalizing behavior	-	-	-		-	-	-		-	-	-		-	-	-	
Y-OQ*®*	-	-	-		-	-	-		-	-	-		-	-	-	

*HED* Heavy Episodic Drinking assessed by AUDIT item 3, cut-off ≥ 2 p. AUDIT total scale cut-off for risk-use was ≥ 6 p. Categorical variable was dichotomized. Genders were analyzed in separate models, and parent-rated predictors were analyzed in separate models. Females *n* = 85, males *n* = 77. Cat = Categorical variable R_N_^2^ = Nagelkerke pseudo R^2^. With correction for multiple testing using the α for significance was set to .01 and CI to 99%

Table 4 presents associations between adolescents’ reported background characteristics and drinking patterns. No significant associations were found with regard to females’ or males’ HED or risk-use.

**Table 4:** Chi-square tests of prediction of females’ and males’ heavy episodic drinking and risk-use of alcohol

	Female HED		Male HED		Female risk-use		Male risk-use	
	*χ*2 (*df*)	*p*	*χ2 (df)*	*p*	*χ2 (df)*	*p*	*χ2 (df)*	*p*
Mother’s country of birth	1.44 (2)	.486	1.36 (2)	.507	2.94 (2)	.230	1.70 (2)	.427
Father’s country of birth	0.57 (2)	.752	1.34 (3)	.718	0.99 (2)	.608	2.23 (2)	.358
Own money to spend	8.16 (6)	.227	13.05(6)	.042	1.89 (6)	.930	13.98 (6)	.030
Family economic standard	1.87 (4)	.759	0.87(4)	.929	6.73 (4)	.151	10.85 (4)	.029

Statistic significance was *p* = .01

### Subscale logistic regressions

Analyses of the subscales constituting externalizing and internalizing behavior revealed that Rule-breaking predicted male HED (*OR* = 1.18, *p* = .010 [*CI* 1.01, 1.39], R_N_^2^ = 0.13) and risk-use *OR* = 1.32, *p* ≤ .001 [*CI* 1.08, 1.61], R_N_^2^ = 0.28), and also female risk-use (*OR* = 1.22 *p* = .003 [*CI* 1.03, 1.44] R_N_^2^ = 0.18).

Prediction analyses using the subscales in Y-OQ®SR showed a somewhat comparable pattern. The subscales Social problems, such as delinquent or aggressive behaviors, predicted female HED (*OR* = 1.23, *p* = .004 [*CI* 1.05, 1.48], R_N_^2^ = 0.21), which also was close to significant for female risk-use (*OR* = 1.16, *p* = .011 [*CI* 1.00, 1.34], R_N_^2^ = 0.12). Furthermore, for the males, the Social problems scale predicted both HED (*OR* = 1.15, *p* = .010 [*CI* 1.00, 1.32], R_N_^2^ = 0.13) and risk-use (*OR* = 1.36, *p* = .001 [*CI* 1.07, 1.72], R_N_^2^ = 0.24).

### Gender similarities

Results from the MRC analyses (not shown in tables) showed no significant differences between male and female prediction slopes for any of the self-rated predictors. On the parent-rated predictors, however, there was a significant gender difference, showing *OR* = 2.41, *p* = .020, indicating that parents’ perception of their adolescent son’s externalizing behavior predicted his risk-use of alcohol to a significantly greater extent than for female adolescents.

## Discussion

### Main findings

The study sample consisted of 162 Swedish alcohol-using adolescents aged 12 − 18 years who were at risk of consolidating antisocial behavior. The aims were to 1. test antisocial behaviors and psychosocial problems as predictors for HED and risk-use, 2. test if parental ratings of the adolescents’ antisocial behaviors predicted the adolescents’ HED and risk-use, and 3. explore if the adolescents’ monthly spending money, perceived family economic standard, delinquent and drinking peers, parental country of birth, gender, and/or age, predicted HED and risk-use of alcohol. Due to earlier research findings of females being more sensitive to peer drinking, we hypothesized that perceived peer drinking would have a larger effect on females’ HED and risk-use compared with that on males. The main findings showed that for at-risk adolescents, perceived peer drinking played significant roles for both females’ and males’ own hazardous drinking, and that female risk-use was predicted by their externalizing behavior. Furthermore, rule-breaking and social problems predicted both HED and risk-use among males, while female rule-breaking only predicted risk-use, and social problems only predicted HED. Finally, the parents’ ratings of externalizing behavior only predicted their sons’ risk-use. Our hypothesis that females’ HED and risk-use would be more affected by peer drinking had to be rejected, since the gender-difference tests (MRC) showed that the perception of peer drinking affected females’ and males’ HED and risk-use similarly.

### Problem behaviors predict HED and risk-use

The PBT theory suggests that multiple problematic influences from individual, behavioral and environmental domains lead to co-occurrence of problem behaviors [11, 13]. In our data, few present problem behaviors predicted HED and risk-use. However, the problems that did showed a similar prediction pattern for both females and males. Future research may consider using other measures, and longer follow-ups to investigate these matters further in order to find promising interventions strategies for hazardous alcohol use.

### Peers influencing alcohol consumption

Further, the PBT notes the importance of deviant peers, and our findings confirm that both females’ and males’ perception of drinking friends predicted their own future drinking similarly for both genders, suggesting that male adolescents may be equally sensitive to peer influence. Peer behavior may have an indirect influence, commonly referred to as the peer influence effect [46], which is proposed to shape social norms and may act as a normative factor. This should, however, be interpreted with caution, as the possibility of a reciprocal relationship with an influence on peer drinking behavior cannot be excluded.

### Age did not predict hazardous drinking

Unlike the study by Thompson, Montgomery [24], we did not find age to be a significant predictor of HED or risk-use, despite our sample’s wide age range. Of course, this may be due to the selection of individuals who had already started to use alcohol; the group prevalence at baseline was 100%.

### Sample considerations

Comparing our scores of externalizing behavior compared with Swedish norm data among 13-18-year olds, our sample appears as an acting-out troubled group. The mean values among females and males were 13.24 (*SD* = 6.92) and 13.77 (*SD* = 7.92) in a normal sample, compared with 22.01 (*SD* = 9.18) and 17.33 (*SD* = 8.47), in our sample. As shown in Table 2, it was also evident that the females in this sample had higher levels of problem behavior than the males, which contradicts the assumption that females in general exhibit lower levels [47, 48]. Keeping in mind that the present sample was included in the original intervention study based on already elevated levels of problem behavior, the females’ levels may be understood in the light of the gender paradox. The paradox assumes that females, who generally exhibit a lower prevalence in externalizing behavior than males, tend to display higher rates of co-occurring problems than males [49]. However, in the present study, the aim was to test if a range of problem behaviors predicted HED and risk-use to a different extent between females and males. We found limited support of this. Despite the females’ significantly higher levels on most of the predictors, the prediction patterns for both genders were largely equal.

Even tough we were unable to test for of adolescent maturation directly in the present sample, maturation can be worthwhile to consider when interpreting the results. Previous research has shown that early menarche is a risk factor for early onset of alcohol use - but only among low-risk girls, especially when the main body of pupils in their school class is girls [50]. Other research has found that advanced pubertal maturation among both adolescent females and males was associated with increased alcohol use [51, 52]. For early maturing females, research suggests that they are more vulnerable to deviant peers, and peers have been found to be a predictor of alcohol use among early maturing, deviant females [52]. Considering our results, it is possible that both the females and males matured early, and therefore had a greater risk of alcohol use, and especially since they also were influenced by alcohol using peers.

### Parents’ problem behavior ratings predicted males drinking and not females

Further, the results showed that parent ratings of externalizing behavior predicted male risk-use. One of the very few studies on the subject showed that parental ratings of youth running away from home, and parental ratings of substance use predicted youth drinking [24], and our findings are reminiscent of those results, but only for males. This indicates that parents may have a better understanding of males’ behavior problems, perhaps due to a common, culturally encoded expectation of male acting-out and alcohol use. Since parental perceptions of adolescents’ behavior are often the reason that parents engage in intervention programs, there is a need to improve the knowledge of the predictive capacity of parental ratings. Lastly, we found that a smaller proportion of females’ mothers than males’ were born outside the Nordic countries. In the previous intervention trial, there was an equal distribution over the three groups [27], although there was an unequal dispersion of non-Nordic mothers between females and males. Since participants originally applied to participate in a study of interventions, males’ non-Nordic mothers may be more willing to undergo parent training, or non-Nordic mothers may experience less concern about their daughters’ behavior than about their sons’.

In summary, the prediction of HED, which puts the user at immediate risk of adversities, and risk-use, which puts the user at risk of both immediate and long-term consequences and dependency, were very similar between genders. Acting-out behaviors, perception of drinking peers, and social problems significantly predicted both HED and risk-use, consistent with the PBT, which suggests that factors from several domains contribute to problem behavior [11].

### Limitations & strengths

One limitation in the present study is its relatively small sample size, and there is a possibility of Type 2 errors. Due to multiple testing of factors predicting HED and risk-use, there is also a possible limitation regarding inflated Type 1 error. By adjusting the alpha level to .01, we attempted to decrease this risk without unacceptably increasing Type 2 risk, but it is possible that there is still a risk of false positive results.

Another limitation concerns the self-reports: there is a possibility of over- and under-reporting, especially of hazardous alcohol use, due to exaggeration and social desirability. However, research on ratings of adolescents’ personality or problem behavior has identified self-ratings as among the most valid methods [53, 54], and self-reported substance use is suggested to be a valid measure [55]. Also, if confidentiality is emphasized for the respondents, self-reports are generally considered reliable [56]. Thus, a bias in the study results should not necessarily be expected due to the use of self-report measures.

Another possible limitation is the use of the AUDIT scale, instead of timeline follow-back, for measuring alcohol use. Timeline follow-back is a measure that should be used to assess daily alcohol use over a longer period of time. By summing all the daily scores, the result shows both variability and magnitude of consumption, and has been proposed to be a precise measure [57]. However, we used the AUDIT scale with pre-defined cut-offs for HED and risk-use as describe above, in accordance with the suggestions of the developers of the timeline follow-back measure: “…when detailed drinking data are either not necessary or not possible to obtain” [p. 861, 57].

This study also has some strengths. Despite being at risk of consolidating a range of problematic behaviors, the target group has not been studied extensively. Longitudinal designs are important, and are probably the most commonly used in prediction studies of adolescents’ hazardous drinking [23, 25, 38, 58, 59], but there is also a need for studies that investigate short-term outcomes, especially in at-risk groups. This short-term follow-up study creates the opportunity to reveal events that occur close in time to the predictor or precursor, and thus the possibility of preventing them. Another strength is that we analyzed females and males separately and with well-studied and validated psychometric instruments. This increases comparability with other national or international studies.

## Conclusions

To conclude, we found that acting-out behavior among female adolescents, and peer drinking in both genders, predicted hazardous drinking behavior. Considering the at-risk sample in the present study, with elevated levels of externalizing behavior, delinquency, psychosocial distress, and alcohol use and its link to peer influence, interventions need to focus partly on peer contextual factors. Also at young ages, it might be difficult to alter behavior and sustain those changes, if the social context contains highly influential features. Since the females in this study had considerably higher levels of externalizing behavior, delinquency, drinking friends, alcohol risk-use, and HED than males, there is reason to believe that at-risk females can be more difficult to treat due to difficulties to reach them at an early stage.

Future research should focus on peer influence by studying the target adolescents’ peers and their actual drinking. Special attention should be on adolescents who are exposed to the risk of maintaining antisocial behavior, in order to develop suitable interventions, including for example peer-influence resistance training.

## Abbreviations

HED: Heavy episodic drinking, i.e., having five drinks or more at one drinking occasion during the last 30 days
MRC: Multiple regression correlation
PBT: Problem-behavior theory
